# Psychological factors associated with exercise self-efficacy in the recipients of an implantable cardioverter defibrillator

**DOI:** 10.1371/journal.pone.0305606

**Published:** 2024-06-25

**Authors:** Pei-Yun Chen, Ching-Yi Chou, Miao-Hsin Lien, Shu-Wen Chen, Shu-Hua Lu, Chyi Lo

**Affiliations:** 1 Department of Nursing, Jen-Teh Junior College of Medicine Nursing and Management, Miaoli County, Taiwan, R.O.C; 2 Department of Nursing, China Medical University Hospital, Taichung, Taiwan, R.O.C; 3 Department of Nursing, Central Taiwan University of Science and Technology, Taichung, Taiwan, R.O.C; 4 School and Graduate Institute of Nursing, China Medical University, Taichung, Taiwan, R.O.C; The University of Mississippi Medical Center, UNITED STATES

## Abstract

**Background:**

Insufficient exercise affects the health of patients who have implantable cardioverter defibrillators (ICD). The purpose of this study was to investigate the correlations between exercise self-efficacy (ESE) and its associated psychological factors in ICD recipients.

**Methods:**

This cross-sectional study included individuals who had undergone ICD implantation at the cardiology department of a medical centre in Taiwan. A face-to-face survey was conducted. The survey questionnaire included questions regarding the participants’ demographics, perceived health (PH), ICD shock–related anxiety (ICD-SRA), self-care self-efficacy (SSE), perceived exercise benefit (PE-benefit), perceived exercise barrier (PE-barrier), and ESE. Data were analysed using SPSS 20.0 Software. Stepwise multiple regression analyses were also performed to evaluate the predictive effects of the aforementioned factors on ESE.

**Results:**

A total of 52 ICD recipients were enrolled. ESE was negatively correlated with ICD-SRA (r = −0.511; *p* < 0.01) and PE-barrier (r = −0.563; *p* < 0.01), but positively correlated with SSE (r = 0.339; *p* < 0.05) and PE-benefit (r = 0.464; *p* < 0.01). The stepwise multiple regression analysis revealed that PE-barrier, PE-benefit, and ICD-SRA effectively predicted ESE in the participants.

**Conclusions:**

ESE may be improved by overcoming PE-barrier, ICD-SRA and enhancing PE-benefit. Consequently, improving ESE may enhance the health benefits of exercise.

## Introduction

Implantable cardioverter defibrillators (ICDs) effectively prevent fatal arrhythmia and reduce the associated mortality rate by 31%. ICDs are a widely used treatment option for preventing sudden death [1]. The American Heart Association (2012 guidelines) recommends ICD as the primary prevention strategy for sudden death in patients with coronary artery disease, dilated cardiomyopathy, and asymptomatic non-sustained ventricular tachycardia with a left ventricular ejection fraction (LVEF) of < 35%. ICD is recommended as the secondary prevention strategy for patients with a history of fatal arrhythmia and a risk of sudden death [2].

Although ICD implantation can prevent sudden death due to fatal arrhythmia, the treatment may have a net negative impact on the recipients’ physical activity and psychological health. Flemme (2001) emphasized that medical conditions warranting an ICD and the consequent lifestyle changes required to accommodate the device diminish the confidence of ICD recipients in performing the activities of daily living, which limits physical activity or exercise [3]. Lemon (2004) reported that approximately 39% of all patients with an ICD avoid physical activity or exercise, primarily because they fear that physical activity would increase their heart rate and risk of ICD shock [4]. Indeed, Berg (2015) reported that only 13% of ICD recipients performed the recommended level of physical activity during the post-implant period (6–12 months) [5]. Another study examining patient-reported outcomes of quality of life and physical activity after ICD implantation found that up to 73.0% of patients in the secondary prevention group admitted that they have limited their physical activity after ICD placement due to fear of electrical shock, leading to a significant reduction in physical activity [6]. Despite this, evidence suggests that physical exercise could reduce the rate of cardiovascular mortality by 20–35% and improve cardiopulmonary function [7]. Furthermore, some studies have reported that physical activity and increased exercise do not increase the risk of shock in individuals with an ICD [8–11]. In short, their findings indicated that the incidence of ICD shock events is not high during exercise, nor was it strongly associated with exercise. Lau (2016) demonstrated that only 2% of all ICD recipients were hospitalised due to an ICD shock; notably, exercise was not a likely cause for any of the incidents [12]. They further stated that early exercise post-implant is safe, effective, and improves perceived exercise self-efficacy (ESE). A study of system review evaluating the effects of exercise training on patients suffering from heart failure who had an ICD suggested that sedentary patients were more prone to ICD shock than those undergoing cardiac rehabilitation and exercise training [8]. Therefore, moderate- to low-intensity exercise in individuals with an ICD does not increase the risk of electric shocks, but rather improves the quality of life and cardiac function.

Self-efficacy is well documented in the literature and is a key determinant for participation in physical activity or exercise by adults with or without heart disease. While the amount of physical activity is influenced by an individual’s physical and medical factors, the level of physical activity is mediated by self-efficacy [13]. According to Bandura’s social cognitive theory, self-efficacy beliefs are influenced by psychological factors, such as affective and cognitive processes, which in turn play crucial roles in shaping behaviour. [14]. Affective processes interact self-efficacy and play significant roles in shaping self-efficacy beliefs. Affective processes involve managing emotions associated with performance and outcomes, such as anxiety and fear, which can hinder performance if poorly managed [15]. Affective processes are therefore closely tied to how individuals cope with stress and adversity and play a vital role in influencing their willingness to engage in physical activity or exercise [16]. A study using the Cardiac Anxiety Questionnaire (CAQ) to assess individuals two years after ICD implantation found that 37% of patients avoided physical activity due to anxiety [17]. Another study has also shown that approximately 44% of patients with an ICD experience shock-related anxiety, which is a fear of being shocked after an activity, and this then leads to a decrease in physical activity [18]. This anxiety or fear can undermine their self-efficacy belief to perform physical activities or exercise.

Self-efficacy is also closely intertwined with cognitive processes, as individuals continuously self-assess their abilities. These processes play a fundamental role in the development, maintenance, and modification of self-efficacy beliefs, all of which influence behaviour [16]. In terms of cognitive processes, the level of perceived health status is a significant factor that modulates the initiation of physical activities. When engaging in healthy behaviours, individuals are influenced more by their perceived health status than objective health assessments [19]. A study investigating the correlation between perceived health status and physical activity levels across 2,587 patients diagnosed with coronary heart disease found a strong association between low self-perceived health and reduced physical activity [20]. Additionally, studies have revealed that an individual’s behaviour can be influenced by personal perceptions of facilitators and barriers, which are themselves reliable predictors of changes in behaviour [21]. Research has shown that these personal facilitators and barriers influence the nature of physical activities performed by patients with heart failure, as they impact the individual’s confidence in their ability to succeed in those activities [22]. Another study revealed that a positive relationship between physical activity situations performed and physical activity self-efficacy in middle-aged and older adults who perceive exercise benefits, coupled with a negative relationship with perceived exercise barriers[23]. Furthermore, self-care efficacy is strongly linked to patients’ capacity to perform daily activities, return to work, and self-care behaviours, and is a crucial factor in adjusting to illness [24].

Although exercise promotes health in ICD recipients and does not increase the probabilities of ICD shock events and anti-tachycardia pacing [25], there is a dearth of literature discussing how exercise self-efficacy factors relate to exercise performance in ICD recipients. Crawford (2013) conducted a correlation study of physical activity among ICD recipients and suggested that more research is needed to identify barriers preventing patient participation in exercises or physical activities [26]. Accordingly, the aims of this study were to examine: (a) the relationship between exercise self-efficacy (ESE) and its psychological factors, such as perceived health (PH), ICD shock–related anxiety (ICD-SRA), self-care self-efficacy (SSE), perceived exercise benefit (PE-benefit), and perceived exercise barrier (PE-barrier) in ICD recipients, and (b) the predictors of exercise self-efficacy in the ICD population. Our hypotheses were as follows: (a) PH, SSE, and PE-benefit are positively associated with exercise self-efficacy in the ICD patient population, (b) ICD-SRA and PE-barrier are negatively associated with exercise self-efficacy in the ICD patient population, and (c) the combination of PH, ICD-SRA, SSE, PE-benefit and PE-barrier can collectively predict exercise self-efficacy in the ICD patient population.

## Methods

### 2.1 Study design and participants

The design of this study was a cross-sectional survey. Individuals who had undergone ICD implantation were recruited from the cardiology department of a medical centre in Taiwan. The study was conducted between March 2018 and September 2020. The department’s medical registry system for ICD case management was used for sampling purposes. The inclusion criteria were as follows: having an ICD for > 3 months, clear consciousness, ability to communicate in Mandarin or Taiwanese, no cognitive dysfunction or mental impairment, no physical activity restrictions, and ability to independently perform the activities of daily living. The exclusion criteria were as follows: having an ICD for < 3 months, inability to communicate in Mandarin or Taiwanese, cognitive or mental disorders, severe physical activity restrictions, inability to independently perform the activities of daily living because of stroke or being bedridden, or heart failure (New York Heart Association Functional Class 4). During follow-up visits at the cardiology department, eligible individuals were requested to participate in a face-to-face questionnaire survey. The participants were informed that communication would be through telephone or email if necessary. Written informed consent was obtained before the survey. Interested individuals had to complete the Mini-Mental State Examination (MMSE) questionnaire before participating in the study. Individuals with MMSE scores of < 23, which indicate cognitive impairment, were excluded from the study. The personal identification information of participants was removed after data collection. Because of the coronavirus disease 2019 pandemic, the number of follow-up visits was limited. This study was approved by the institutional review board of the study centre (approval no. CMUH106-REC-021).

### 2.2 Survey instruments

#### 2.2.1 Participants’ demographics and medical history

Data regarding the participants’ demographics and medical history were obtained through the self-report survey and the department’s medical records. Demographic data included age, sex, marital status, education level, and work status. Medical data included comorbidities, LVEF before ICD implantation, history of cardiopulmonary resuscitation, and number of ICD shocks.

#### 2.2.2 Perceived Health (PH)

The participants were asked about their perceived overall health status. The responses were rated on a 5-point Likert-type scale with the endpoints ranging from 1 (*very bad*) to 5 (*very good*). Higher scores indicated better PH. This questionnaire is often used in community surveys when evaluating self-reported health status, and the consensus is that it serves as a useful summary of how patients perceive their overall health status. This is a powerful predictor of clinical study outcomes in a wide range of disease areas [27].

#### 2.2.3 ICD Shock-related Anxiety (ICD-SRA)

The Florida Shock Anxiety Scale (FSAS), which was developed by Kuhl (2006), was used to evaluate the level of ICD-SRA. This scale comprises a total of 10 items. The responses are rated on a 5-point Likert-type scale (1 = *strongly disagree*, 2 = *disagree*, 3 = *neutral*, 4 = *agree*, and 5 = *strongly agree*). Exploratory factor analysis revealed a two-factor structure with item loadings. The FSAS exhibits good reliability (Cronbach’s α = 0.91; split-half reliability = 0.92; test-retest total score = 0.79; *p* < 0.01). Regarding discriminant validity, the total score on the FSAS strongly correlated (r = −0.65; *p* < 0.01) with that on the Multidimensional Fear of Death Scale [28]. Higher scores indicate higher ICD-SRA levels.

#### 2.2.4 ICD Self-care Self-Efficacy (SSE)

ICD self-care self-efficacy was assessed using the Self-Efficacy Expectations after ICD Implantation Scale established by Dougherty [29], which helps evaluate self-efficacy expectations and behaviours necessary for managing common problems. The scale comprises questions regarding seven areas of concern: preventive care, healthcare providers, partnerships, daily activities, physical changes, emotional challenges, and ICD defibrillation. The scoring method is as follows: 1 = *not at all*, 2 = *approximately 30% confidence*, 3 = *approximately 50% confidence*, 4 = *approximately 70% confidence*, and 5 = *approximately 100% confidence*. This scale was chosen for its reliability and internal consistency (Cronbach’s alpha = 0.93). The total scores on this scale correlated positively with the scores on General Self-Efficacy instruments (r = 0.48, p < 0.01), and negatively with anxiety as measured by the State–Trait Anxiety Inventory (r = -0.61, p < 0.01) [29], supporting good concurrent validity. Higher scores are associated with greater self-efficacy.

#### 2.2.5 Perceived exercise benefit (PE-benefit) and Perceived exercise barrier (PE-barrier)

After reviewing the literature on the benefits of, and barriers preventing, exercise as perceived by older adults, we developed two scales to evaluate the levels of PE-benefit and PE-barrier in ICD recipients. Both scales included specific factors affected by the operation of the defibrillator. The scales for evaluating PE-benefit and PE-barrier comprised a total of 8 and 12 items, respectively. The scoring method was as follows: 1 = *strongly disagree*, 2 = *disagree*, 3 = *neutral*, 4 = *agree*, and 5 = *strongly agree*. These modified scales were validated by experts, and the index of content validity (CVI) for evaluating PE-benefit and PE-barrier were 0.96 and 0.94, respectively. The modified scales for this study also exhibited good internal consistency (Cronbach’s alpha = 0.70 and 0.85, respectively).

#### 2.2.6 Exercise Self-Efficacy (ESE)

The Scale of Exercise Self-Efficacy was established by Resnick in 1995 as a self-report instrument to measure exercise self-efficacy. The scale consists of 9 items that assess an individual’s confidence in their ability to overcome barriers and maintain regular exercise habits [30]. This scale exhibits good internal consistency (Cronbach’s alpha = 0.92), test-retest reliability, and concurrent validity with other measures of physical activity behaviour. Due to specific factors related to physical activities affected by the defibrillator, four questions were added to the scale. The scoring method for each item was as follows: 1 = *not at all*, 2 = *approximately 30% confidence*, 3 = *approximately 50% confidence*, 4 = *approximately 70% confidence*, and 5 = *approximately 100% confidence*. The scale has been validated by experts (CVI = 0.96) and exhibited good internal consistency (Cronbach’s alpha = 0.87). Higher total scores indicate a higher level of exercise self-efficacy.

### 2.3 Statistical analysis

Categorical variables are presented as numbers and percentages, whereas continuous variables are presented as means and standard deviations. Pearson correlation analysis was performed using SPSS for Windows (version 20.0) to investigate the correlations of ESE with PH, ICD-SRA, SSE, PE-benefit, and PE-barrier in ICD recipients. Multiple linear regression was conducted to explore the predictors of exercise self-efficacy. The variables that were significantly (*p* < 0.05) correlated with ESE were defined as independent variables. The predictive effects of the independent variables on ESE were further analysed using stepwise multiple linear regression. Statistical significance was set at *p* < 0.05.

## Results

The demographic information of the participants is summarized in Table 1. This study included a total of 52 adults (proportion of men, 70.6%; mean age, 53 years; range, 32–81 years). Most participants had a senior high school or associate degree. The mean LVEF before ICD implantation was 37.4%, indicating poor left ventricular function. Nearly half of the individuals (46.2%) were diagnosed with more than three cardiovascular diseases, suggesting more cardiovascular-related co-morbidity in this population. Of the individuals, 82.7% had experienced shock due to cardiopulmonary resuscitation, indicating a high proportion of secondary prevention. However, nearly 70% reported having no experience of shock caused by the ICD device.

**Table 1:** Participant demographics (N = 52).

	n/ mean	%/SD
Age*	53.0	12.4
Sex		
Male	36	70.6
Female	15	29.4
Missing	1	1.9
Marital status		
Never married	10	19.2
Married	35	67.3
Divorced	3	5.8
Widowed	4	7.7
Education level		
None or elementary school	10	19.2
Junior high school	6	11.5
Senior high school	11	21.2
Associate’s degree	13	25.0
Bachelor’s degree	10	19.2
Graduate degree	2	3.8
Work status		
Working	17	32.7
Nonworking	35	67.3
No. of Cardiovascular-related co-morbidity*	2.37	1.07
< 3	28	53.8
> 3	24	46.2
LVEF pre-implant (%)*	37.4	4.26
Cardiopulmonary resuscitation		
Never	8	15.4
Yes	43	82.7
Missing data	1	1.9
ICD shock		
Never	36	69.2
Yes	15	28.8
Missing data	1	1.9
Exercise self-efficacy score		
Low	32	61.5
High	20	38.5

*Data are presented in terms of mean and standard deviation (SD) values. LVEF, left ventricular ejection fraction; ICD, implantable cardioverter defibrillator.

The distribution of participants’ scores across the study’s variables is presented in Table 2. The participants’ ESE scores ranged from 17 to 42 (mean score = 23.4 ± 3.3). When calculated by dividing the total score by the number of questions, the mean score for the ESE was only 1.8. Individuals who scored above the average constituted a high-score group, whereas those who scored below average constituted a low-score group. Most individuals were included in the low-score group (61.5%; Table 1), indicating the prevalence of lower ESE among the ICD population. The participants’ PH scores ranged from 2 to 4 (total score = 5; average score = 3.5). The mean ICD-SRA score was 35.2, which indicated moderate to high levels of ICD-SRA. Most individuals had PE-barrier scores above the average; when the total score was divided by the number of questions, the mean score was as high as 4.07. This indicated high levels of PE-barrier among the participants (Table 2).

**Table 2:** Distribution of the participants’ scores in the study variables.

	Mean	SD	Min	Max
Exercise self-efficacy	23.4	3.3	17.0	42.0
Perceived health	3.5	0.6	2.0	4.0
ICD shock–related anxiety	35.2	4.1	24.0	46.0
Self-care self-efficacy	39.6	4.9	27.0	51.0
Perceived exercise barrier	48.8	3.8	36.0	54.0
Perceived exercise benefit	28.2	2.3	16.0	31.0

The results of the Pearson correlation analysis are summarized in Table 3. ESE was shown to be significantly correlated with most of the variables listed in the study (*p* < 0.05), with the exception of demographics and PH. ESE was negatively correlated with ICD-SRA (r = −0.511; *p* < 0.01) and PE-barrier (r = −0.563; *p* < 0.01) but positively correlated with SSE (r = 0.339; *p* < 0.05) and PE-benefit (r = 0.464; *p* < 0.01).

**Table 3:** Correlations between the study variables in ICD recipients (N = 52).

	ESE	PH	SRA	SSE	PE-barrier	PE-benefit
ESE	1	0.268	−0.511**	0.339*	−0.563**	0.464**
PH		1	−0.003	0.078	0.107	0.325*
ICD-SRA			1	-0.454**	0.497**	−0.310*
SSE				1	−0.304*	0.378**
PE-barrier					1	−0.333*
PE-benefit						1

*p < 0.05 and

**p < 0.01.

ESE, exercise self-efficacy; PH, perceived health; SRA, shock-related anxiety; SSE, self-care self-efficacy; PE-barrier, perceived exercise barrier; and PE-benefit, perceived exercise benefit.

We further evaluated the predictive effects of ICD-SRA, SSE, PE-barrier, and PE-benefit on ESE. In a stepwise regression analysis, ESE served as the dependent variable while the independent variables were those previously shown to be significantly correlated with ESE (*p* < 0.05). In the first step of the analysis, the model included only PE-barrier as the independent variable, and the observed results were significant (F = 23.152; *p* < 0.01). In the second step, the model included only PE-benefit, and the results were also shown to be significant (F = 16.512; *p* < 0.01). Finally, ICD-SRA was included in the regression model, and the results were significant (F = 13.093; *p* < 0.01). Further analysis using a multiple linear regression model effectively explained approximately 42% of the variance observed on exercise self-efficacy (F = 13.09, P < 0.001). In the stepwise regression analysis, ESE was effectively predicted by PE-barrier (β = −0.297), PE-benefit (β = 0.380), and ICD-SRA (β = −0.201). The following equation was used for the prediction model (Table 4):

ESE = 34.31 + (−0.297) × PE-barrier + 0.380 × PE-benefit + (−0.201) × ICD-SRA

**Table 4:** Stepwise regression model for predicting exercise self-efficacy (N = 52).

Adjusted R^2^	F	Model	Β	Unstandardized Coefficients Std. Error	Standardised Coefficients Beta	T	P*-* value	95% CI for B
0.42	13.09**	(Constant)	34.31	7.82		4.39	.000	(18.58, 50.04)
		PE-barrier	−0.297	0.109	−0.346	−2.74	.009	(−0.52, 0.08)
		PE-benefit	0.380	0.162	0.270	2.34	.024	(0.05, 0.71)
		ICD-SRA	−0.201	0.099	−0.255	−2.03	.047	(−0.40, 0.00)

*p < 0.05 and

**p < 0.01.

PE-barrier, perceived exercise barrier; PE-benefit, perceived exercise benefit; ICD, implantable cardioverter defibrillator; and SRA, shock-related anxiety.

## Discussion

### ✧ Characteristics of ICD population

It has been demonstrated that ICD devices improve survival rates of patients who are at high risk of sudden death from cardiac-related issues. The collected data in this study supported key information in determining the best practices for ICD therapies. The demographics and medical characteristics within our ICD population male-dominated, middle-aged and elderly adults, more cardiovascular-related comorbidities, and poor left ventricular EF. These characteristics were consistent with a review from the NCDR ICD registry [31]. The high proportion of indications from secondary prevention found in our data was the only difference in a guideline of indications for ICD implantation. This difference is mainly due to our national health care policies. Therefore, most of our participants experienced ICD and shock due to cardiopulmonary resuscitation.

### ✧ Exercise self-efficacy and physical activity among ICD population

There is a paucity of research examining the relationship between ICD patients’ self-efficacy regarding exercise and psychological factors. Our study is one of the few to provide preliminary evidence on the link between ESE and its psychological factors among ICD recipients. The results of this study suggest that ICD recipients have low ESE, implying that ICD implantation might have a significant impact on a patient’s self-efficacy regarding exercise, thereby reducing their physical activities in daily life. Some studies have already supported the connection between low ESE and individuals with cardiovascular disease and chronic illness [32,33]. Pozehl et al. (2007) investigated the correlation between physical activity and exercise self-efficacy in patients with heart failure by using the Exercise Self-Efficacy Scale. The study found a significantly positive correlation between physical activity and exercise self-efficacy (r = 0.32, *p* = 0.045), suggesting that greater self-efficacy in heart failure patients led to increased physical activity [34]. Although there was a lack of direct correlational data between the level of patient’s ESE and physical activity among ICD recipients, some studies have shown that these patients have low confidence in undertaking regular physical activities [3,5,35]. Alternatively, another study evaluating the safety and efficacy of a home-based walking program for the ICD population identified a strong correlation between ESE and exercise behaviours [12]. Overall, these results imply that ESE levels and exercise behaviour are positively associated with one another, and that developing ESE beliefs is important for initiating and maintaining an exercise regimen [36].

### ✧ Exercise self-efficacy of psychological factors among ICD population

In our study, ICD recipients were shown to have moderate to high levels of ICD-SRA. Furthermore, ICD-SRA was found to be negatively correlated with ESE, which implies that higher levels of ICD-SRA are consistent with lower levels of ESE, and thus lower levels of confidence to engage in sports. This finding is in line with those of similar studies [18,37–39]. Unlike patients with other cardiac diseases, ICD recipients have a specific ICD-SRA. The perceived risk of shock and accelerated heart rates during exercise markedly affects the exercise behaviours of these individuals, thus limiting their engagement in sports and intense physical activities [37–39]. Sweeting (2017) evaluated the impact of ICD implantation on physical activity levels in ICD recipients and showed that individuals with high levels of ICD-SRA also exhibited low levels of confidence in performing physical activity [35]. They further explained that being anxious about potential ICD shocks was the only factor preventing them from meeting physical activity guidelines. Sears (2018) investigated the correlation between ICD-SRA and physical activity in a total of 2,770 patients who either had an ICD or were receiving cardiac resynchronisation therapy with a defibrillator. In most patients, the levels of ICD-SRA increased after the patients experienced a shock, leading to a subsequent reduction in physical activity levels. As the number of shock events experienced increased, overall anxiety increased, and physical activity levels considerably decreased [40]. Although exercise is safe and effective, these individuals now overlook its benefits due to the fear of ICD-related adverse events, resulting in low confidence levels regarding exercise participation.

In this study, self-care self-efficacy (SSE) was shown to be positively correlated with ESE. Although evidence from studies on the direct relationship between SSE and ESE among people with ICD is sparse, some studies have shown that self-care confidence mediates the relationship between cognition and self-care behaviours [24], as well as patients’ physical and psychological health [41]. Additionally, this finding corroborates that of a systematic review, conducted by Kavradim (2020), which showed that patients with heart disease who have higher levels of SSE exhibited better disease management and exercise adherence [42].

Most research has shown that perceived health competence is strongly associated with physical activity [19,20,43]. A higher perceived state of health is correlated to increased physical activity levels, making health perception a significant predictor of physical activity. However, some studies have argued that no associations exist between an individual’s perceived health status and their physical activity [44,45]. Indeed, during this study, no strong correlation between ESE and PH was found. This might be because PH was evaluated based on a single question, which was done to streamline the survey and reduce the burden on participants. Thus, future studies should include appropriate instrumentation and more comprehensive questioning.

### ✧ Predictive model

Regression models have rarely been used to predict ESE. In this study, ESE served as a dependent variable in a stepwise regression model. Our regression model demonstrated that ESE can be predicted by the variables of PE-barrier, PE-benefit, and ICD-SRA, with the proportion of variance explained by reaching up to 42% despite the limited sample size. Consequently, the effect size is considerably large at 0.72. A power analysis using G*Power 3.1.7 indicated a high level of statistical power of 0.99.

In our study, the results have shown that ESE was negatively correlated with PE-barrier and positively associated with PE-benefit in ICD patients. These findings were consistent with previous research examining the relationship between PE-barrier, PE-benefit, and ESE across various diseases [23,26,46]. The results in this present study suggest that ESE levels are positively associated with exercise behaviour, whereas PE-benefit and PE-barrier have a positive and negative impact, respectively, on ESE. Also, both were significant predictors of behavioural changes.

Furthermore, negative emotions such as fear and anxiety have been shown to be determining factors for physical activity or exercise levels in ICD recipients; thus, such emotions may help predict their level of physical activity [15,47]. In this study, ICD-SRA was identified as an important predictor of ESE, which is consistent with the findings of other relevant studies [35,39,40]. In a study on ICD recipients’ confidence in performing physical activity, ICD-SRA was the only important factor that affected an individual’s level of physical activity [35]. Additionally, the European Society of Cardiology indicates that, when it comes to cardiac rehabilitation, device-induced shock is the only concern for patients who have an ICD [48]. The present study goes further in that the data reflects the strong correlation between ICD-SRA and PE-barrier, and this in turn may predict low levels of ESE. Thus, individuals with high levels of ICD-SRA experience a high PE-barrier, and therefore lack confidence when it comes to performing exercise.

Although SSE was positively correlated with ESE, it was excluded from the regression prediction model. This could be because SSE is correlated primarily with disease-related self-care behaviours, and exercise comprises only a small part of the total self-care regime. Self-care consists of multiple levels, including psychological adjustment. Because of this dilution effect, SSE may not be a good predictor of ESE in ICD recipients.

### Study limitations

The present study had some limitations. Firstly, this was a single medical centre study with a limited sample size, and the coronavirus disease pandemic led to there was a reduction in follow-up visits. Additionally, the study did not encompass all areas of the country. Thus, these findings may not be comparable to other centres that may have different patient demographics, treatment protocols, or healthcare infrastructure. Future studies should be conducted with a larger number of cases from multiple centres to increase the inferential value of the findings. Secondly, the cross-sectional correlation design of this study did not allow us to understand the long-term changes in the physical activity behaviour of the ICD population. Future studies may adopt a longitudinal study design to monitor changes in physical activity levels over time. Thirdly, we evaluated PH based on participants’ responses to a single question; thus, the depth of assessment for PH was not comprehensive. Future studies should include comprehensive questioning to better interrogate the level of self-perceived health within the population. Finally, this study utilised a quantitative approach. However, ESE is a subjective psychological concept with nuanced degrees of manifestation; hence future studies should combine suitable qualitative and quantitative methods to comprehensively explore the factors associated with ESE in ICD recipients.

## Conclusions and implications

There were three primary findings of this study. Firstly, there was a negative correlation observed between ESE and both PE-barrier and ICD-SRA. Secondly, ESE was positively correlated with SSE and PE-benefit. Thirdly, the predictors (PE-barrier, PE-benefit, and ICD-SRA) collectively accounted for up to 42% of the variance in ESE analysis. The high variance explained by the model suggests that these variables have a meaningful impact on ESE. Overall, these findings imply that interventions aimed at reducing PE-barrier, lowering ICD-SRA, and emphasizing PE-benefit, can contribute to increased exercise self-efficacy among the population of ICD patients.

In particular, specific ICD-SRA serves as a key predictor for differentiating the ICD population from other patients with other heart diseases. This anxiety can cause individuals to neglect the advantageous effects of exercise due to fear of adverse events from the ICD device. It is crucial for healthcare professionals to recognize early the unique anxiety associated with ICD devices and the potential for electric shocks. Healthcare professionals must equip patients with accurate information and address misconceptions about exercise to reduce ICD-SRA. They should also assess and understand the possible perceptions that ICD recipients may have about exercise to identify potential factors contributing to PE-barrier and PE-benefit. For future clinical interventions, personalised care plans coupled with counselling may enhance PE-benefit among ICD recipients. This, in turn, could assist in overcoming PE-barrier. Consequently, patients may become more motivated to engage in regular exercise, which is crucial for their overall health and well-being.
